# Standardizing lymphangiography and lymphatic interventions: a preclinical in vivo approach with detailed procedural steps

**DOI:** 10.1186/s42155-023-00364-z

**Published:** 2023-03-30

**Authors:** Feng Pan, Thuy D. Do, Niclas Schmitt, Dominik F. Vollherbst, Markus Möhlenbruch, Parham Tinoush, Alexander Brobeil, Vitali Koch, Götz M. Richter, Philippe L. Pereira, Hans U. Kauczor, Christof M. Sommer

**Affiliations:** 1 Clinic of Diagnostic and Interventional Radiology, Heidelberg University Hospital, Heidelberg, Germany; 2 Department of Radiology, Union Hospital, Tongji Medical College, Huazhong University of Science and Technology, Wuhan, China; 3 Department of Neuroradiology, Heidelberg University Hospital, Im Neuenheimer Feld 400, 69120 Heidelberg, Germany; 4 General Pathology and Pathological Anatomy, Heidelberg University Hospital, Im Neuenheimer Feld 224, 69120 Heidelberg, Germany; 5 Pathological Institute, NCT Tissue Bank, Heidelberg University Hospital, Im Neuenheimer Feld 224, 69120 Heidelberg, Germany; 6 Institute for Diagnostic and Interventional Radiology, Frankfurt University Hospital, Theodor-Stern-Kai 7, 60590 Frankfurt Am Main, Germany; 7 Clinic of Diagnostic and Interventional Radiology, Klinikum Stuttgart, Stuttgart, Germany; 8 Clinic for Radiology, Minimally-Invasive Therapies and Nuclearmedicine, SLK-Kliniken GmbH, Heilbronn, Germany; 9 Clinic of Neuroradiology, Stuttgart Clinics, Katharinenhospital, Kriegsbergstrasse 60, 70174 Stuttgart, Germany

**Keywords:** Lymphography, Lymphatic Intervention, Postoperative Lymphatic Leakage, Animal Model, Preclinical Training

## Abstract

**Purpose:**

To present a preclinical in vivo approach for standardization and training of lymphangiography and lymphatic interventions using a pictorial review.

**Materials and methods:**

Different lipiodol- and gadolinium-based lymphangiography and lymphatic interventions were performed in twelve (12) landrace pigs with a mean bodyweight of 34 ± 2 kg using various imaging and guiding modalities, similar to the procedures used in humans. The techniques used were explicitly introduced and illustrated. The potential applications of each technique in preclinical training were also discussed.

**Results:**

By applying visual, ultrasonography, fluoroscopy, CT, cone-beam CT, and/or MRI examination or guidance, a total of eleven techniques were successfully implemented in twelve pigs. The presented techniques include inguinal postoperative lymphatic leakage (PLL) establishment, interstitial dye test, five types of lymphangiography [incl. lipiodol-based translymphatic lymphangiography (TL), lipiodol-based percutaneous intranodal lymphangiography (INL), lipiodol-based laparotomic INL, lipiodol-based interstitial lymphangiography, and interstitial magnetic resonance lymphangiography (MRL)], and four types of percutaneous interventions in the treatment of PLL [incl. thoracic duct embolization (TDE), intranodal embolization (INE), afferent lymphatic vessel sclerotherapy (ALVS), and afferent lymphatic vessel embolization (ALVE)].

**Conclusion:**

This study provides a valuable resource for inexperienced interventional radiologists to undergo the preclinical training in lymphangiography and lymphatic interventions using healthy pig models.

## Introduction

The lymphatic system is a one-way vessel system in the body besides the cardiovascular system, which regains proteins and interstitial fluid, regulates the immune response, and absorbs lipids from the intestine (Mallick and Bodenham 2003). It originates from the interstitial spaces with blind ends of the capillary lymphatic vessels, absorbing tissue fluid to form lymph fluid. Then, the lymph fluid flows in the lymphatic vessels through several lymph nodes to obtain lymphocytes and plasma cells, converges into the thoracic and right lymphatic ducts, and finally enters the cardiac vascular circulation through the bilateral jugular angles. However, our understanding of lymphatic physiology and pathology is limited, and the availability of lymphangiography and lymphatic interventional techniques is not prevailed worldwide (Weiss and Liddell 2020). So, there are still insufficiencies in diagnosing and treating lymphatic diseases caused by damage to the lymphatic circulation system. For instance, surgery may damage the lymphatic vessels, resulting in the iatrogenic loss of these nutrient fluids, or so-called “postoperative lymphatic leakage” (PLL), which is a difficult-to-treat and potentially life-threatening complication (Schild et al. 2013; Lv et al. 2017). Untreated PLL can cause continuous nutrient loss and significantly prolong postoperative recovery time, eventually resulting in death events (Lv et al. 2017; Sommer et al. 2020). Traditional conservative treatments (e.g., total parenteral nutrition, somatostatin, etc.) have poor effects on high-output PLL (> 1000 ml/d); moreover, surgical repairs for PLL (e.g., lymphatic vessel ligation, lymph-venous anastomosis, etc.) have high complication and mortality rates of as high as 30% and 25% respectively, (Schild et al. 2013; Pan et al. 2020a, b, c). So, it is essential to propagate the roles of lymphatic imaging and interventions, which can be used not only in the lymphatic diagnosis but also in the minimally invasive treatment of PLL.

Fortunately, lymphatic diseases are increasingly coming into focus. By generation, different techniques of lymphangiography have been developed, such as translymphatic lymphangiography (TL), intranodal lymphangiography (INL), and contrast-enhanced interstitial magnetic resonance lymphangiography (MRL), to achieve adequate imaging of the lymphatic system (Pieper et al. 2019; Pieper et al. 2020). These imaging techniques can delineate lymphatic circulation, accurately assess lymphatic diseases, and guide further percutaneous interventions (Guermazi, Brice et al. 2003, Inoue et al. 2016; Pieper et al. 2019; Pan et al. 2020a, b, c). For example, if PLL was identified, two major types of percutaneous interventions can be chosen: one is leakage embolization [e.g., thoracic duct embolization (TDE), intranodal embolization (INE), etc.], the other is sclerotherapy [e.g., afferent lymphatic vessel sclerotherapy (ALVS), etc.] (Pan et al. 2020a, b, c; Sommer et al. 2021). Choosing appropriate interventions based on prior lymphangiography can effectively cure this severe postoperative complication (Pan et al. 2020a, b, c; Sommer et al. 2021).

So far, understanding the pathophysiology of the impaired lymphatic circulation system and making an optimized clinical management plan for lymphatic diseases are still essential (Weiss and Liddell 2020). However, because of the relatively little clinical need, only a few centers have the conditions to carry out these procedures. Even worse, the technical diversity and complexity of variable lymphangiography and lymphatic interventions increase the difficulty of promoting these techniques. In order to overcome these issues, it is beneficial to establish a standardized approach in a non-human model. To date, a few studies elucidated pigs' lymphatic structures, resembling humans (Dori et al. 2014; Ito and Suami 2015; Kraitchman et al. 2017). Hence, they have the potential to become an ideal objective as a non-human model. In this center, we have performed further exploration from PLL establishment to lymphangiography and lymphatic interventions in healthy landrace pigs. Herein, we systematically summarize and elaborate our standardized approach from the animal preparation to the lymphatic imaging and interventions step by step in the pig model, which might facilitate the in vivo lymphatic research, the preclinical training, and propagation of lymphatic radiology in the future.

## Materials and methods

### Animal preparation

This investigational protocol involving animals was in accordance with the “Guide for the Care and Use of Laboratory Animals” and the institutional regulations of scientific working on animals (AZ 35–9185.81/G-3/18) (Singh et al. 2011). The Institutional Animal Care and Use Committee approved all research procedures in this study. Twelve (12) healthy German landrace female pigs with a mean body weight of 38 kg ± 4 (range: 32-42 kg) served as study animals. Standardized pig preparation followed: general anesthesia was initiated with an intramuscular cocktail consisting of 10 mg ketamine, 6 mg azaperone, and 0.4 mg midazolam per kilogram of body weight; then, intubation was performed, and general anesthesia was subsequentially maintained with isoflurane inhalation; central venous catheters were inserted afterwards (Sommer et al. 2015, Pan, Vollherbst et al. 2020). All following procedures and examinations were performed under general anesthesia.

### Case collections

Apart from representations of PLL establishments at the groin and interstitial dye tests, this study predominantly illustrated two classifications of lymphatic-related techniques in the swine model: lymphangiography and lymphatic interventions. In lymphangiography, three access routes were tested in the pig model: interstitial, translymphatic vessel, and intranodal (Pieper et al. 2019; Pan et al. 2020a, b, c; Sommer et al. 2021). For the visualization of the lymphatic system in lymphangiography, two types of contrast were used: lipiodol [Lipiodol® (Ethiodized Oil) Injection, Guerbet, Roissy, France] for X-ray opacification and gadolinium contrast [Primovist® (gadoxectic acid 0.25 mmol/ml), Bayer Vital, Germany] for magnetic resonance imaging (MRI) enhancement. In sum, the techniques of lymphangiography in this study included: 1. lipiodol-based TL; 2. lipiodol-based percutaneous INL; 3. lipiodol-based laparotomic INL; 4. lipiodol-based interstitial lymphangiography; 5. interstitial MRL. The lymphatic interventions in this study include sclerotherapy and embolization, both of which are used to treat lymphatic leakage (Pieper et al. 2019; Pan et al. 2020a, b, c; Sommer et al. 2021). The techniques of lymphatic interventions had: 1. thoracic duct embolization (TDE); 2. intranodal embolization (INE); 3. afferent lymphatic vessel sclerotherapy (ALVS); 4. afferent lymphatic vessel embolization (ALVE). We aim to elucidate and illustrate the explicit techniques in our pig model and discuss the potential application of each method in animal research or preclinical training. The procedures are summarized in Table 1.

**Table 1:** A summary of different procedures in pig models

Sequence	Types	Pig No	Access route	Operative site	Contrast	Equipment	Sclerosant	Embolized agent	Objective
1	PLL establishment	1;2;3;4;5;6		Groin					Inguinal PLL establishment
2		1;2;3;4	Interstitial	Foot	Patent Blue V				Interstitial dye test
3	Lymphangiography	1	Translymphatic	Upper /lower limb	Lipiodol	DSA/CT			Lipiodol-based TL
4	Lymphangiography	7;8	Intranodal	Neck/groin	Lipiodol	US/DSA/CT			Lipiodol-based percutaneous INL
5	Lymphangiography	4;9	Intranodal	Mesentery	Lipiodol	DSA/CT			Lipiodol-based laparotomic INL
6	Lymphangiography	2;3;5;6;10	Interstitial	Foot/hand	Lipiodol	DSA/CT			Lipiodol-based interstitial lymphangiography
7	Lymphangiography	11;12	Interstitial	Foot	Gadoxetate disodium	MRI			Interstitial MRL
8	Embolization	7;8	Translymphatic	Abdomen		DSA/CT		NBCA/coils	TDE
9	Embolization	10	Intranodal	Neck		DSA/CT		NBCA	INE
10	Sclerotherapy	5	Translymphatic	Thigh		CT	95% Ethanol		ALVS
11	Embolization	1	Translymphatic	Thigh		DSA/CT		NBCA	ALVE

*Abbreviations: PLL* Postoperative lymphatic leakage, *DSA* Digital subtraction angiography, *CT* Computed tomography, *US* Ultrasonography, *MRI* Magnetic resonance imaging, *NBCA* N-butyl-2-cyanoacrylate, *TL* Translymphatic lymphangiography, *INL* Intranodal lymphangiography, *MRL* Magnetic resonance lymphangiography, *TDE* Thoracic duct embolization, *INE* Intranodal embolization, *ALVS* Afferent lymphatic vessel sclerotherapy, *ALVE* Afferent lymphatic vessel embolization

### Radiological equipment

The examining modalities of ultrasonography (US), fluoroscopy/digital subtraction angiography (DSA)/cone-beam computed tomography (CBCT), conventional computed tomography (CT), and magnetic resonance imaging (MRI) were respectively performed on a US system (Acuson S2000, Siemens Healthcare, Germany), a DSA system (Artis Zee; Siemens Healthineers, Germany), a multi-detector CT scanner (SOMATON Definition Flash; Siemens Healthineers, Germany), and a 1.5-Tesla MRI scanner (Aera; Siemens Healthineers, Germany), respectively, following the same parameters for human beings (Kortes et al. 2014; Pieper and Schild 2018; Pan et al. 2020a, b, c; Pan et al. 2020a, b, c; Pieper et al. 2020).

### Histopathological examinations of lymph nodes

To explore the histopathological changes of lymph nodes after exposure to lipiodol and glue, after lipiodol-based intranodal lymphangiography and INE, the pigs were sacrificed immediately or 6 days later using intravenous injection of 20 ml potassium chloride 7.5%. Then, the punctured and embolized lymph nodes at the groin or neck were harvested under the fluoroscopy guidance and fixed in 4% buffered paraformaldehyde. Afterward, the samples were paraffin-embedded for further pathological analysis. From each paraffin block, two adjacent Sects. (3-4 μm) were obtained and stained according to the standard hematoxylin and eosin (HE) protocol (Sommer et al. 2015).

## Results

### Inguinal PLL establishment

Inguinal PLL is one of the most common PLLs after surgeries or interventions, such as subcutaneous tumor resection, inguinal lymphadenectomy, and femoral arterial/venous catheterization (Pan, Richter et al. 2022). So, we can choose the groin region as the target for the PLL establishment. The subcutaneous distribution of lymphatic vessels at the groin simplifies the PLL establishment.

**Fig. 1:**
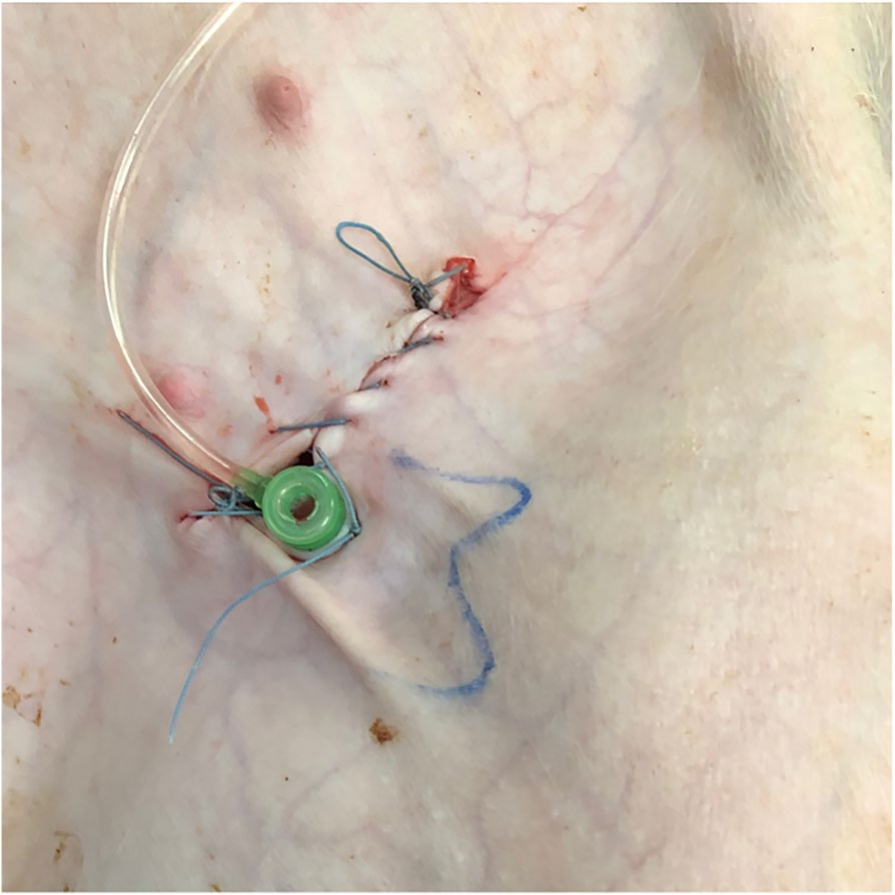
PLL establishment in a healthy pig. After a transverse incision at the left groin, a 6-French vascular sheath accessed the left femoral artery under direct vision. Afterwards, the incision was sutured. The image was from *Pig No.3*. Abbreviation: PLL, postoperative lymphatic leakage

*Materials and Methods.* To simulate a surgical procedure potentially leading to a PLL, a transverse surgical cut down with about 5 cm length is carried out along with the groin in order to expose the femoral artery and vein. Then, a 6-French sheath (Radifocus™ Introducer II, Terumo, Tokyo, Japan) is accessed into the exposed common femoral artery, simulating the femoral arterial catheterization. Hereafter, the incision is sutured with the fixation of the arterial sheath (Fig. 1). The established PLL is directly characterized by dye or contrast extravasation outside the lymphatic system in interstitial dye test or any lymphangiography mentioned below (Kawasaki et al. 2013; Yoshimatsu et al. 2013; Pieper et al. 2020).

### Interstitial dye test

The interstitial dye test can be used not only for the optical visualization of subcutaneous lymphatic vessels and lymph nodes before lymphangiography but also for the indirect identification of PLL (Pieper et al. 2019; Li et al. 2021, Pan, Richter et al. 2022). Lymphatic vessels can be demonstrated visually following the interstitial injection of a diffusible dye, such as Patent Blue V (100 mg/4 ml, Guerbet, Roissy, France), which is the most satisfactory lymphatic dye. In vivo, it diffuses rapidly and clears rapidly with reasonable safety. It is typically introduced prior to the traditional translymphatic lymphangiography (e.g., transpedal) in humans or intranodal lymphangiography in rabbits (Matsumoto et al. 2019; Pieper et al. 2019; Pan et al. 2020a, b, c, Pan, Richter et al. 2022). A positive result of the interstitial dye test is one of the main diagnostic criteria of PLL (Pan et al. 2020a, b, c; Li et al. 2021, Pan, Richter et al. 2022).

**Fig. 2:**
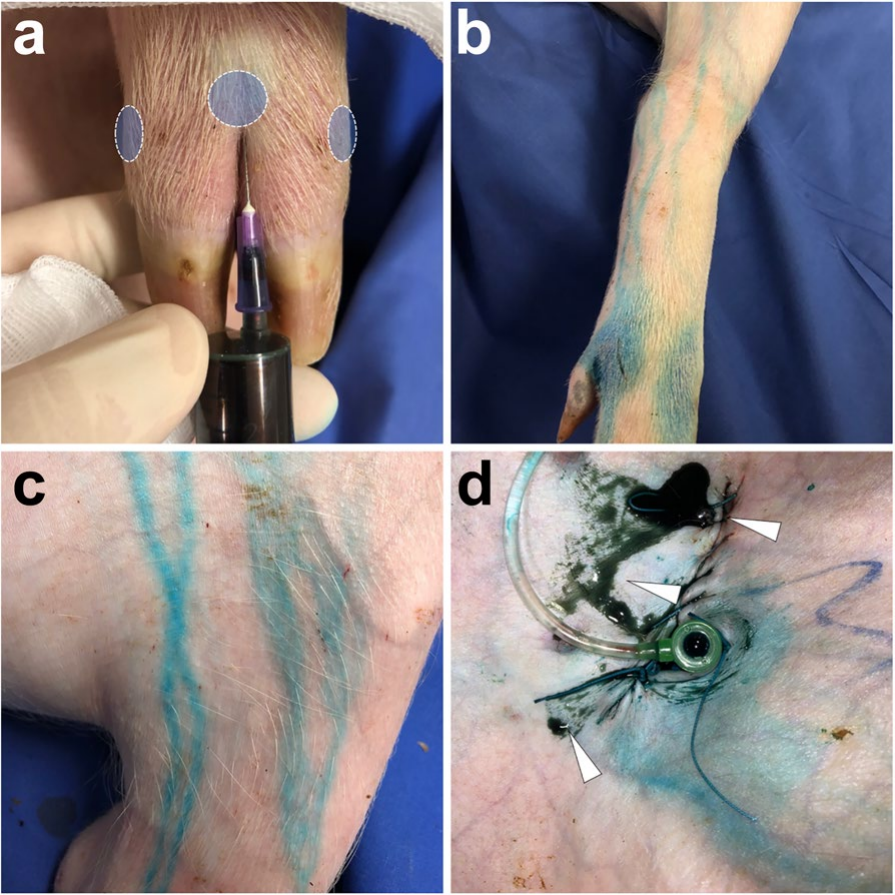
Interstitial dye test with the PLL identification. Fifteen minutes after the interdigital (*imaginary circle*) injection of Patent Blue V (**A**) at the right foot (1 ml per each interdigital space), the subcutaneous lymphatic vessels at the lower limb (**B**) and thigh (**C**) were dyed blue color which could be visually observed. With more time, PLL could be identified by the blue dye extravasation (*white arrowheads*) from the incision around the arterial sheath (**D**). Images were from *Pig No. 3*. Abbreviation: PLL, postoperative lymphatic leakage

*Materials and Methods.* 1 to 2 ml of a 1:1 mixture of Patent Blue V and 1% lidocaine is injected into each dorsal interdigital space on both hands and feet. 15 to 20 min later, the lymphatic vessels can be visually identified in the upper and lower limbs. If an inguinal PLL exists, the blue dye extravasation from the incision can be observed. Figure 2 shows an example of a positive interstitial dye test in a PLL pig model.

### Lipiodol-based lymphangiography

In the 1960s, ultra-fluid lipiodol became an ideal contrast agent in lymphangiography (Dargent et al. 1962). Its insoluble feature made it difficult to diffuse out of the lymphatics (Dargent et al. 1962). Two accesses for the lipiodol injection can be chosen: translymphatic and intranodal routes, corresponding to TL and INL techniques. In TL, the lymphatic vessels on the dorsal foot are separated and punctured by a small needle. After the fixation of the needle, lipiodol is manually injected with a velocity between 0.1 to 0.5 ml/min to successively opacify the lymphatic vessels and lymph nodes from the lower leg to thigh, pelvis, retroperitoneal region, cisterna chyli, and thoracic duct under fluoroscopy (Pieper et al. 2019). However, cannulation is difficult and time-consuming because the peripheral lymphatic vessels are fragile. After the 2010s, INL gradually replaced this method (Nadolski and Itkin 2012). During INL, a thin needle is punctured into the medulla of the inguinal lymph node under ultrasonography guidance. Afterwards, the injected lipiodol can enter the efferent lymphatic vessels through the connected medulla of the lymph node. The technical difficulty of INL is lower than that of TL, but it is still challenging to ensure the precise location of the needle tip. The procedure is similar when performing TL or INL in the pig model. After that, we will introduce the details of the lipiodol-based lymphangiography techniques which can be carried out in pig models. Besides, we also test the feasibility of interstitial lipiodol lymphangiography, which has never been reported before.

### Lipiodol-based TL

*Materials and Methods.* Before lipiodol-based TL, interstitial Patent Blue V lymphangiography must be performed to visualize the subcutaneous lymphatic vessels at the upper and lower extremities. 15 to 20 min after interdigital Patent Blue V injection, incise the dorsal skin at the upper or lower limb along with the dyed lymphatics. Then, separate an accessible lymphatic vessel as a target for the cannulation. Afterwards, puncture the target lymphatic vessel using a 26-gauge trocar needle (Neoflon™ Pro IV Catheter, BD, Franklin Lakes, New Jersey) and fix it with a suture and sterile tapes. Then, connect the trocar to a syringe with an infusion tube and manually conduct the lipiodol with the recommended injection velocity between 0.1 to 0.5 ml/min, depending on the injecting resistance.

**Fig. 3:**
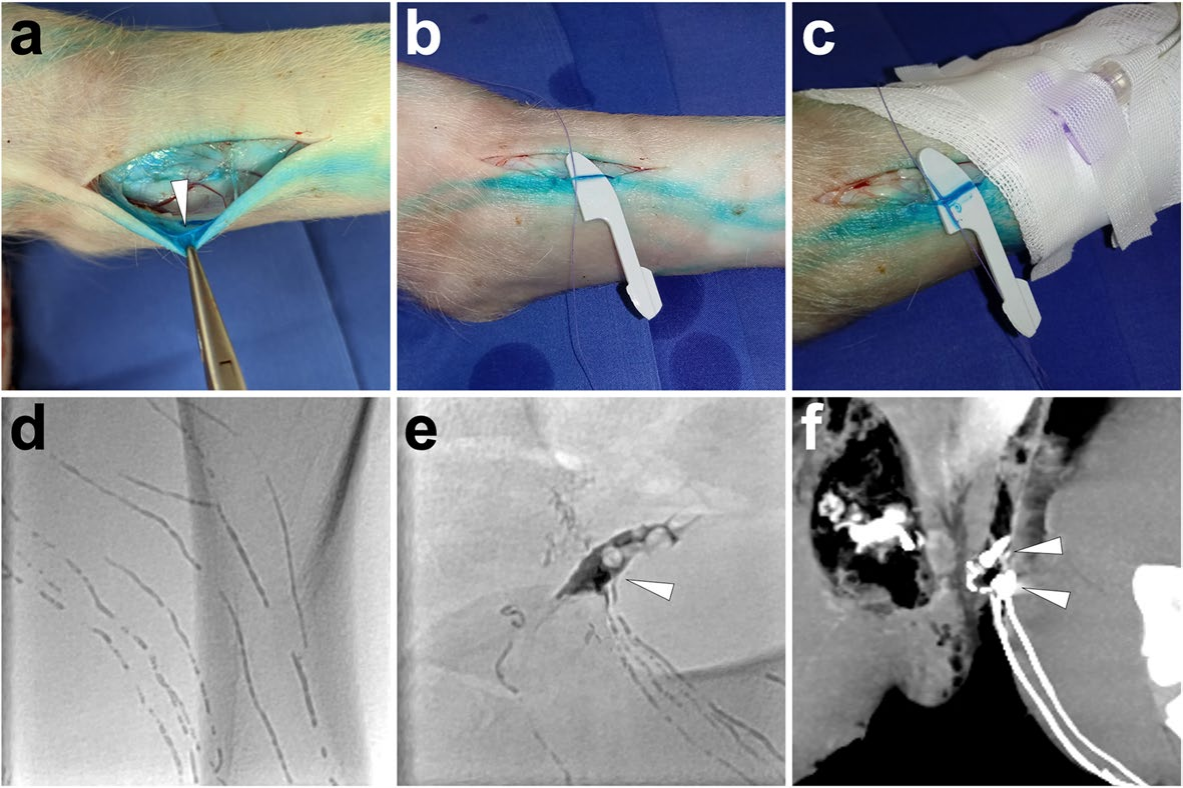
Illustration of lipiodol-based TL in a PLL pig model. Fifteen minutes after Patent Blue V injection at feet, along with the dye, incise the skin at the left lower limb to identify an accessible lymphatic vessel (*white arrowhead*) as the target for cannulation (**A**). Then, separate the targeted lymphatic vessel with a plastic blade and a suture (**B**). Afterwards, access the lymphatic vessel with a 26-gauge trocar needle, which was later fixed and connected to an infusion tube **(C)**. When manually injecting the lipiodol, linear lymphatic vessels in the lower limb were opacified under fluoroscopy (**D**). The lipiodol extravasation (*white arrowhead*) could be fluoroscopically observed at the left groin with lymphatic vessel disruptions (**E**). In the coronal MIP image of the sequential post-lymphangiography CT, lipiodol extravasation (*white arrowheads*) could also be delineated (**F**). Images were from *Pig No. 1*. Abbreviations: TL, translymphatic lymphangiography; PLL, postoperative lymphatic leakage; MIP, maximum intensity projection; CT, computed tomography

*Imaging.* From the initiation of lipiodol injection, intermittent fluoroscopy is used to monitor the lymphatic opacification dynamically. If the opacification of lymphatics is not observed but with lipiodol extravasation around the trocar, reperform the cannulation again. The lipiodol injection persists until the left or right jugular vein angle is opacified. If the lipiodol-based TL is performed in the PLL model, evident opacified lipiodol extravasation at the groin around the arterial sheath can be found under fluoroscopy. After accomplishing TL, the needle is removed, and the wound is sutured. At last, the sequential CT examination is performed with 3-dimensional reconstruction with the same parameters for human beings (scan parameters: 120kv, adaptive current, and B30f iterative reconstruction) to present a more detailed anatomy of the lymphatic system, which is the so-called post-lymphangiography CT (Pan et al. 2020a, b, c). In Fig. 3, we demonstrate a typical process of TL with cannulation at the lower limb in a PLL pig model.

### Lipiodol-based percutaneous INL

*Materials and Methods.* Ultrasonography examination of the subcutaneous lymph nodes is performed to identify suitable puncture targets. Theoretically, subcutaneous lymph nodes at different pig parts can be targeted, such as the neck, groin, axillary, etc., depending on the objective of lymphangiography. Then, puncture the target lymph node with a 22-gauge Chiba needle (BD Spinal needle, BD, Franklin Lakes, New Jersey) under ultrasonography guidance and place the tip of the needle within the central hyperdense portion of the lymph node (indicating medulla). We recommend an acute angle for puncture due to the stability of the needle under a long subcutaneous tract. Besides, there is a caveat: do not puncture the lymph node more than once to avoid peripheral lipiodol leakage. Next step, remove the stylet and connect the trocar to a syringe with an infusion tube. To confirm the proper position of the needle tip, a small amount of lipiodol (0.1–0.5 ml) should be manually injected as a test under fluoroscopy. Signs of successful cannulation of the lymph node are the gradual opacification of efferent lymphatic vessels and lack of perinodal lipiodol extravasation under fluoroscopy. Afterwards, manually inject the lipiodol with a velocity of 0.1–0.5 ml/min under intermittent fluoroscopy. If the lymph node and its efferent lymphatics are not clearly identified or lipiodol extravasation is evidently observed, finely adjust the position of the needle within the lymph node or re-puncture an adjacent lymph node.

**Fig. 4:**
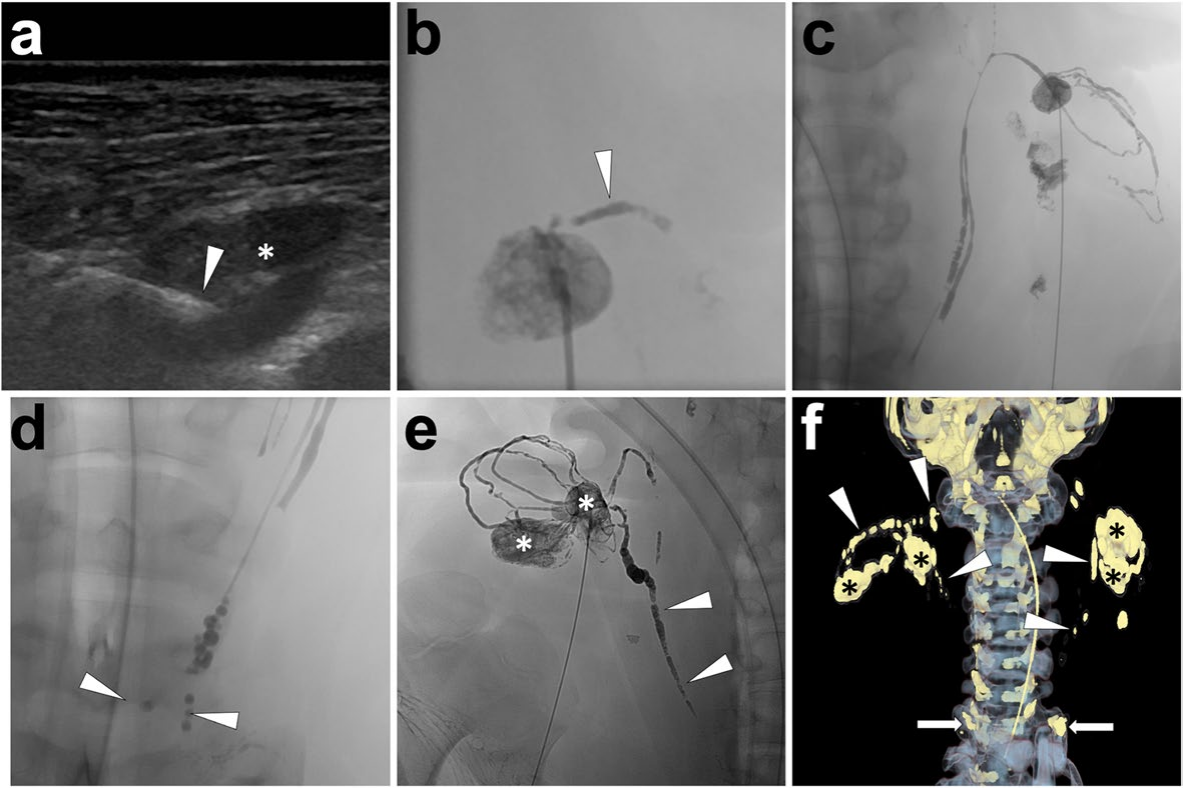
Illustration of lipiodol-based cervical INL in a pig model. In this case, lipiodol-based cervical INL was introduced. Under ultrasonography guidance, the tip of the needle (*white arrowhead*) was advanced until reaching the center of the target lymph node (*) (**A**). An efferent lymphatic vessel (*white arrowhead*) at the left neck was opacified after a small amount of lipiodol injection (**B**); then, continue the lipiodol injection. Over time, lipiodol gradually shifted towards the left jugular vein angle over time (**C**). The lipiodol injection ceased when oil-dropped lipiodol entered the left brachiocephalic vein (*white arrowheads*; **D**). As the same, the lymph nodes (*), efferent lymphatic vessels, and right lymphatic duct (*white arrowheads*) at the right side of the neck were gradually opacified after the lipiodol injection (**E**). After INL, remove the needles and carry out the volume rendering reconstruction of post-lymphangiography CT to demonstrate the three-dimensional lymphatic structure, such as lymph nodes (*), lymphatic vessels (*white arrowheads*), and bilateral confluences of jugular venous angles (*white arrows*; **F**). Images were from *Pig No. 7*. Abbreviation: INL, intranodal lymphangiography; CT, computed tomography

*Imaging.* Fluoroscopy is used to dynamically monitor the opacification of the lymphatic system from the initiation of lipiodol injection. End the lipiodol injection when the left or right jugular vein angle is opacified based on the access route. Then, remove the needle and carry out the post-lymphangiography CT examination, as the same as done in lipiodol-based TL. In Fig. 4, we exemplify a cervical INL procedure in a PLL pig model.

### Lipiodol-based laparotomic INL

*Materials and Methods*. As an alternative to percutaneous inguinal INL, laparotomic INL can be another way to demonstrate the cisterna chyli and thoracic duct. Firstly, linearly cut down the midline of the anterior abdominal wall and expose the mesentery under laparotomy. Then, detect the mesenteric lymph nodes grossly, which look like translucent bubbles. Puncture one of the lymph nodes using a 22-gauge needle and locate the needle tip at the center of the lymph node. Afterwards, manually inject the lipiodol at a rate of 0.1 ml/min under fluoroscopy to identify the proper position of the needle tip, similar to percutaneous INL.

**Fig. 5:**
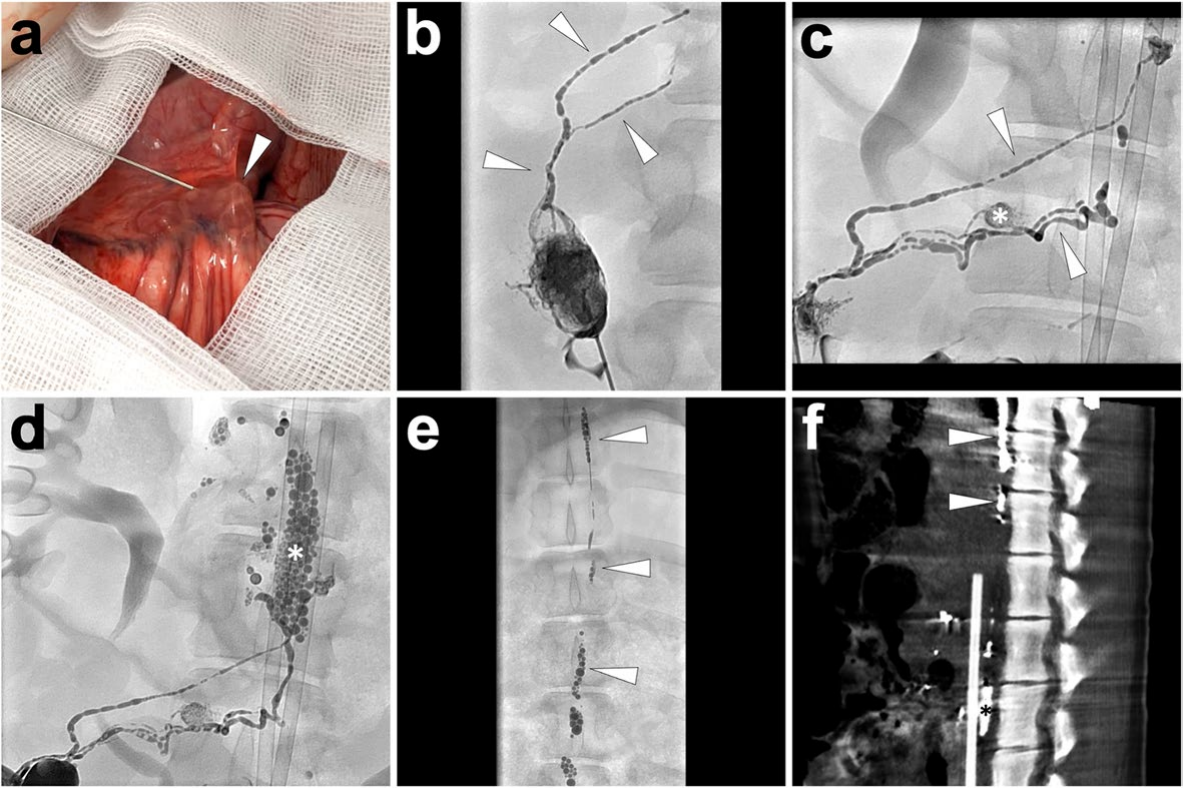
Illustration of lipiodol-based laparoscopic INL in a pig model. After laparotomic exposure of the mesentery, the translucent lymph node (*white arrowhead*) was punctured using a 22-gauge Chiba needle (**A**). Under fluoroscopy monitoring, a small amount of lipiodol (1 ml) was first injected. The opacification of the targeted lymph node and efferent lymphatic vessels (*white arrowheads*) indicated an ideal needle position (**B**). Afterwards, continuous injection of lipiodol visualized the mesenteric lymphatics (*white arrowheads*; **C**), mesenteric lymph nodes (*; **C**), cisterna chyli (*; **D**), and thoracic duct (*white arrowheads*; **E**). The MIP image of the following post-lymphangiography CT presented the details between lymphatic structures such as cisterna chyli (*) and thoracic duct (*white arrowheads*) to the surrounding tissue (**F**). Images were from *Pig No. 4*. Abbreviations: INL, intranodal lymphangiography; MIP, maximum intensity projection; CT, computed tomography

*Imaging.* Under dynamical fluoroscopy, monitor the lymphatic flow from mesenteric lymphatic vessels to the cisterna chyli, which can be consistently observed at L2/L3 level (about the renal hilum level in pigs). Over time, the lipiodol flow opacified the thoracic duct until the left jugular venous angle. Then, stop the lipiodol injection and implement the post-lymphangiography CT. Figure 5 illustrates a detailed process of lipiodol-based laparotomic INL in a pig model.

### Lipiodol-based interstitial lymphangiography

Whether performing lipiodol-based TL or INL, accurate puncture or intubation is the key to ideal imaging. Technically, puncturing small lymphatic vessels or lymph nodes is challenging and time-consuming. Visualizing the peripheral lymphatic system based on subcutaneous interstitial injection of contrast can avoid such difficulties in operations. To test the feasibility of this method, we perform the lipiodol-based transpedal interstitial lymphangiography in our pig model.

**Fig. 6:**
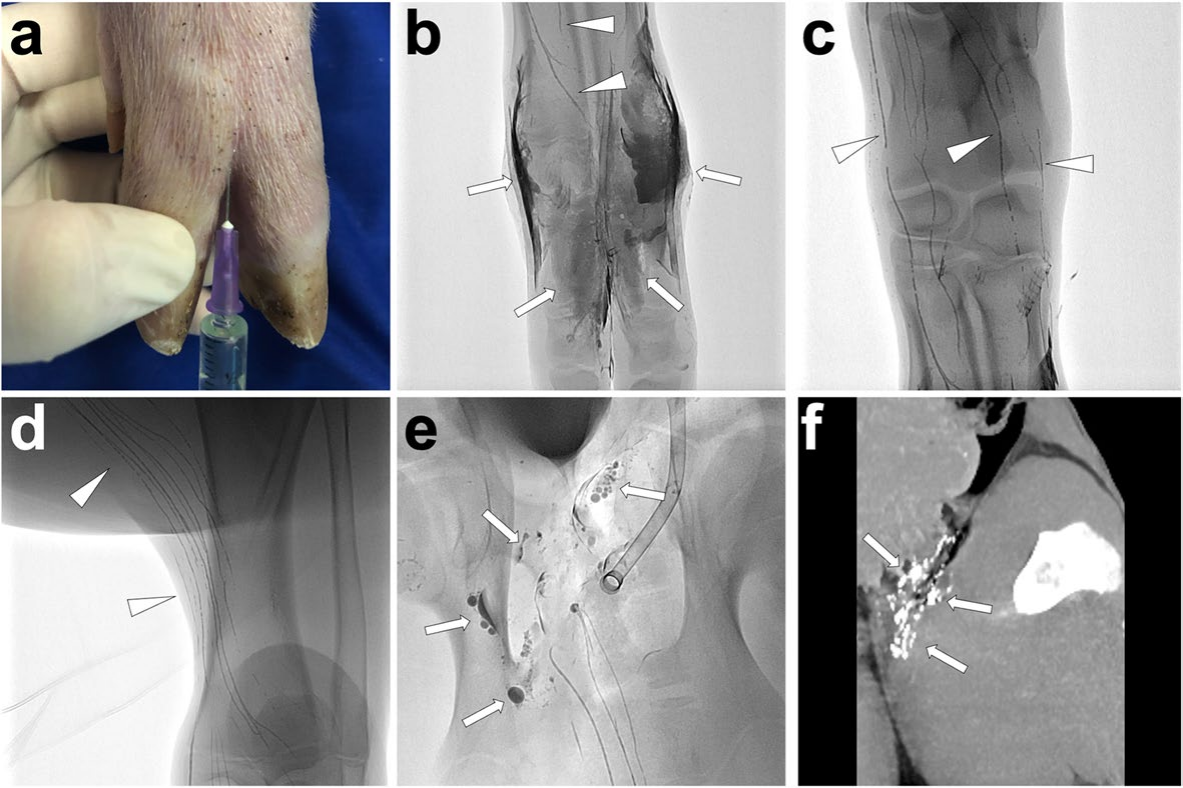
Illustration of lipiodol-based interstitial lymphangiography in a pig model. First, inject 2 ml lipiodol at each interdigital space of the left foot, similar to the interstitial dye test (**A**). After 15 min, the interstitial (*white arrows*) and surrounding lymphatic vessels (*white arrowheads*) were opacified under fluoroscopy (**B**). Over time, the lymphatic vessels (*white arrowheads*) at the ankle (**C**) and lower leg (**D**) were visually opacified under fluoroscopy. When the lipiodol reached the left groin region, evident extravasation of lipiodol (*white arrows*) was observed with the disruption of lymphatic vessels, indicating a typical PLL diagnosis (**E**). The coronal MIP image of the sequential post-lymphangiography CT demonstrated the subcutaneously sporadic distribution of lipiodol (*white arrows*) at the left groin (**F**). Images were from *Pig No. 10*. Abbreviations: PLL, postoperative lymphatic leakage; MIP, maximum intensity projection

*Materials and Methods.* Similar to the Interstitial dye test, 2 ml lipiodol is injected into each interdigital space at the dorsal feet. *Imaging.* Fifteen minutes later**,** monitor the lymphatic flow of the lipiodol under dynamic fluoroscopy. Over time, we can observe the opacification of the lymphatic vessels from feet to lower leg, thigh, and groin. However, this method did not achieve the opacification of the concentrical lymphatic ducts, such as cisterna chyli, thoracic duct, and right lymphatic duct. If this procedure was performed in a PLL pig model, evident lipiodol extravasation at the groin around the incision could also be observed under fluoroscopy or sequential CT scan. As an example, we illustrate a case in Fig. 6.

### Interstitial MRL

Because water-soluble iodinated contrast agents may rapidly shunt from lymphatic vessels into venous capillaries, it is difficult to visualize the lymphatic circulation under fluoroscopy and CT scan. However, gadolinium-based contrast provides a feasible way to visualize the peripheral lymphatic system under an MRI scan by interstitial injection, which is well tolerated by patients (Suga et al. 2003; Dori et al. 2014; Mazzei et al. 2017; Pieper et al. 2020). Preliminary clinical trials have also shown that interstitial MRL can accurately evaluate the lymphatic system (Mazzei et al. 2017; Pieper et al. 2020). It is consistent with traditional lipiodol-based lymphangiography and can also provide clinically useful information for diagnosing pathological lymphatic abnormalities, anatomic variation, and lymphatic fistulas (Pieper et al. 2020). In this study, we performed this technique in a pig model using the same operation in humans.

**Fig. 7:**
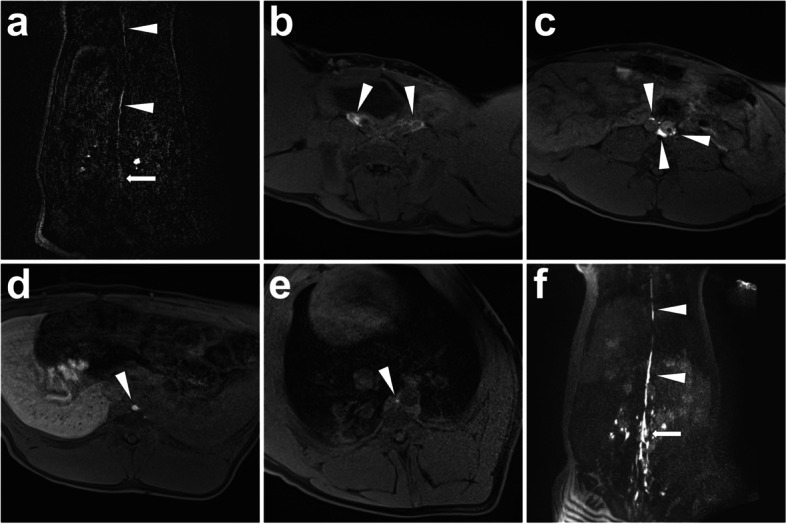
Illustration of interstitial MRL in a pig model. After interstitial injection of gadoxetate disodium at the bilateral foot dorsal, a dynamic coronal 3D-TWIST scan was performed. Thirty minutes later, the definite thoracic duct (*white arrowheads*) started from cisterna chyli (*white arrow*) was visualized (**A**). Afterwards, a 3D-FLASH scan was performed, which presented the continuous lymphatic network surrounding bilateral iliac arteries and veins (*white arrowheads*; **B**) to retroperitoneal lymphatic vessels (*white arrowheads*; **C**) and thoracic duct (*white arrowhead*; **D**, **E**). Coronal 3D-FLASH MIP image demonstrated a clear structure of retroperitoneal lymphatic vessels, cisterna chyli (*white arrow*), and proximal thoracic duct (*white arrowheads*) (**F**). Images were from *Pig No. 11*. Abbreviations: MRL, magnetic resonance lymphangiography; 3D-TWIST, three-dimensional time-resolved angiography with interleaved stochastic trajectories; 3D-FLASH, three-dimensional fast low angle shot; MIP, maximum intensity projection

*Materials and Methods.* Gadolinium contrast is injected into three dorsal interdigital spaces at each foot with a recommended total dose of 0.025 mmol/kg body weight. *Imaging.* After contrast injection, the three-dimensional time-resolved angiography with three-dimensional time-resolved angiography with interleaved stochastic trajectories (3D-TWIST) scan is performed to dynamically monitor the lymph flow until the complete visualization of the thoracic duct (Fig. **7**). Afterwards, the three-dimensional fast low angle shot (3D-FLASH) scan is sequentially carried out to visualize lymphatic vessels and neighboring structures with better image qualities (Fig. 7). Typical 3D-TWIST imaging parameters are as follows: repetition time (TR)/echo time (TE) 3 ms/1 ms; flip angle, 25°; bandwidth 1300 Hz/pixel; matrix, 320*240 mm^2^; field of view, 300*450 mm^2^; section thickness, 1.2 mm, 88 partitions; and isotropic voxel size, 1.2*1.2*1.2 mm^3^; acceleration factor, 2; partial Fourier transform 80%; the percentage of the center of k-space (A) is 10% and the percentage of data points in the periphery of k-space (B) is 15%; the sequence has a temporal resolution of 3.9 s, interpolated to 1.95 s; a total of 9 dynamics are acquired; the total acquisition time is 22 s. Typical imaging parameters of 3D-FLASH were as follows: TR/TE 3 ms/1 ms; flip angle, 25°; bandwidth 1300 Hz/pixel; matrix, 320*240 mm^2^; field of view, 300 mm*450 mm; section thickness, 1.2 mm; 88 partitions; isotropic voxel size, 1.2*1.2*1.2 mm^3^; acceleration factor, 2; Partial Fourier transform 80%; the total acquisition time is 22 s.

### Treatment of PLL

After confirmation of PLL from lymphangiography, the afferent lymphatic vessels and lymph nodes can be considered as the targets for different percutaneous interventions, including thoracic duct embolization (TDE), intranodal glue embolization (INE), and percutaneous lymphatic vessel sclerotherapy/embolization (AVLS/ALVE) (Pan et al. 2020a, b, c; Sommer et al. 2021). Either embolization or sclerotherapy can seal the PLL if the treating target is close to the leakage site because it avoids persistent leakage from collaterals. However, in the pig models, we can train different interventions on the assumption of PLL at variable regions. In the following, we will introduce four typical types of lymphatic interventions performed in our pig models

### Thoracic duct embolization (TDE)

The TDE is one of the most described interventions in previous literature, which is always used to treat postoperative chylothorax or chylous ascites resulting from the ruptured thoracic duct. In the TDE procedures, a microcatheter is advanced into the thoracic duct proximal to the extravasation site from percutaneous puncture. Afterwards, trans-microcatheter embolization can be performed with coils and/or glue (Cope and Kaiser 2002, Boffa et al. 2008, Mittleider et al. 2008, Itkin, Kucharczuk et al. 2010, Yannes et al. 2017, Majdalany et al. 2018, Reisenauer et al. 2018, Jun, Hur et al. 2021). Considering the possibility of ectopic embolism of pulmonary arteries when singularly using the diluted glue, coils can be suggested to release before glue embolization in order to intercept the glue flow forwarding lungs. From previous studies, TDE has shown outstanding efficiency in treating postoperative chylothorax, with a clinical success rate of 84.6% to 100.0%; still, the technical success rate was low at 37.5% because of the difficulty of thoracic duct catheterization (Cope and Kaiser 2002, Boffa et al. 2008, Mittleider et al. 2008, Itkin, Kucharczuk et al. 2010, Yannes et al. 2017, Majdalany et al. 2018, Reisenauer et al. 2018, Jun, Hur et al. 2021). The most common access for thoracic duct microcatheterization is the trans-cisterna chyli route, apart from the trans-thoracic duct route, which is a bail-out if the cisterna chyli is not well developed (Cope and Kaiser 2002, Boffa et al. 2008, Mittleider et al. 2008, Itkin, Kucharczuk et al. 2010, Yannes et al. 2017, Majdalany et al. 2018, Reisenauer et al. 2018, Jun, Hur et al. 2021). Empirically, a thoracic duct size of more than 2 mm is considered accessible (Cope and Kaiser 2002). However, no matter which access route is chosen, it is necessary to obtain skilled operation from sufficient training. Fortunately, the pig model can become an excellent objective for interventionists to practice TDE procedures in preclinical training.

**Fig. 8:**
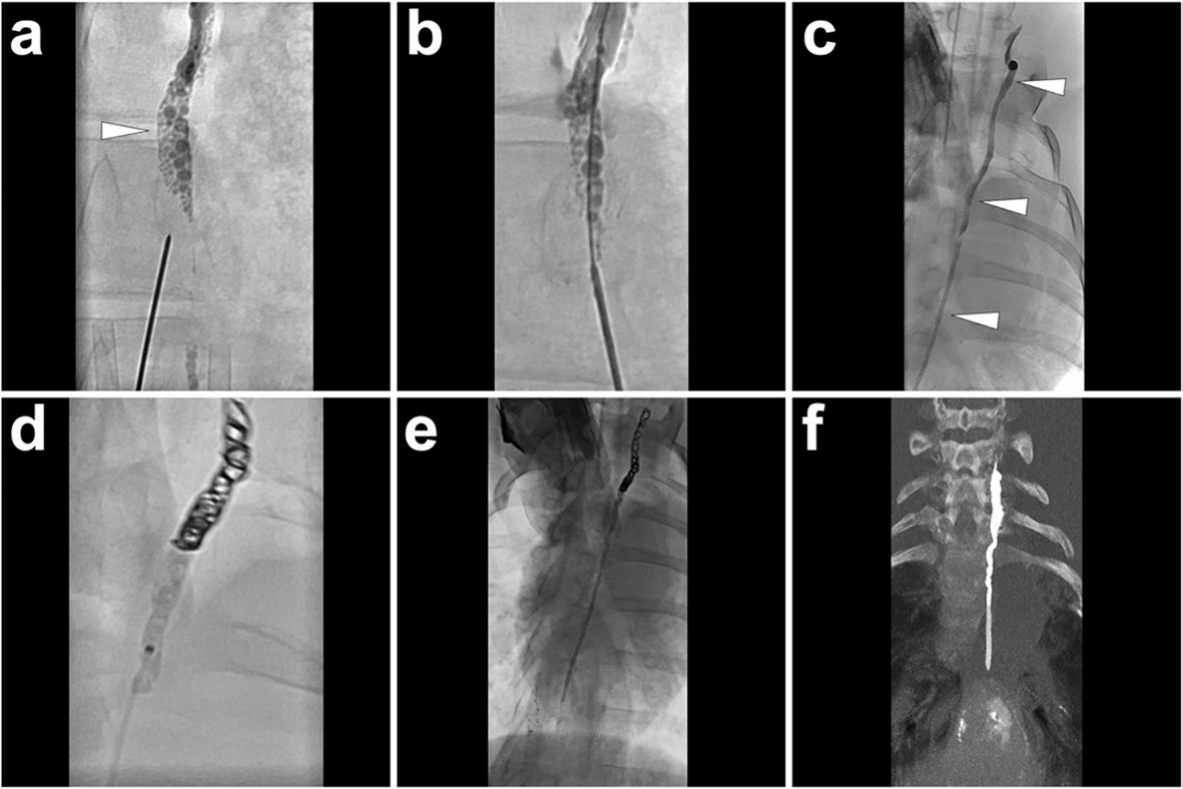
Illustration of TDE using coils and sequential NBCA/lipiodol mixture embolization. A 22-gauge Chiba needle was punctured into the cisterna chyli (*white arrowhead*) under fluoroscopy (**A**). Then, remove the stylet and coaxially exchange a 0.018-inch guidewire into the thoracic duct (**B**). Coaxially advance a microcatheter into the thoracic duct and conduct an angiography to delineate the complete thoracic duct (*white arrowheads*) using the water-soluble iodinated contrast (**C**). Afterwards, advance the microcatheter into the distal part of the thoracic duct with reference to the angiographic image and release four coils (4 mm*14 cm, Nester® Embolization Coil, Cook Medical, Bloomington, USA) followed by glue injection (in this pig, 4 ml 1:2 NBCA/lipiodol mixture was used) with the simultaneous withdrawal of the microcatheter under fluoroscopy (**D**). After the accomplishment of embolization, perform the fluoroscopy (**E**) and CT scan (MIP image, **F**) to demonstrate the embolized thoracic duct presenting as a linear opacity. Images were from *Pig No. 8*. Abbreviations: TDE, thoracic duct embolization; NBCA, N-butyl-2-cyanoacrylate; CT, computed tomography; MIP, maximum intensity projection

*Materials and Methods.* Before the procedure, eligible lipiodol-based lymphangiography (e.g., TL, inguinal INL, etc.) should be performed to visualize cisterna chyli and thoracic duct well. After choosing an accessible cisterna chyli or thoracic duct, percutaneously advance a 22-gauge Chiba needle (Neff Percutaneous Access Set, Cook Medical, Bloomington, USA) from the anterior abdominal wall into the target duct under fluoroscopy with both the bull-eye and paralleled flat-detector monitoring. After entering the lymphatic duct, remove the stylet and advance a 60 cm 0.018-inch guidewire (Neff Percutaneous Access Set, Cook Medical, Bloomington, USA) through the trocar. Manipulate the guidewire into the thoracic duct; then, remove the trocar and coaxially introduce the plastic stiffening cannula (Neff Percutaneous Access Set, Cook Medical, Bloomington, USA). Afterwards, coaxially exchange the guidewire to a more extended 0.018-inch guidewire (HiWire® Hydrophilic Wire Guide 180 cm, Cook Medical, Bloomington, USA). Then, remove the plastic stiffening cannula and advance a 2.5-French microcatheter (Cantata® Microcatheter 135 cm, Cook Medical, Bloomington, USA). Thereafter, remove the guidewire and perform an angiography by injecting the water-soluble contrast (Iomeron®-400, Bracco Imaging SpA, Milan, Italy) to check whether the microcatheter was located in the thoracic duct. After confirming the right catheterization in the thoracic duct, advance the microcatheter to the distal part of the thoracic duct with reference to the prior angiography; then, embolization can be initiated. After coils embolization, sequential glue embolization is followed. The N-butyl-2-cyanoacrylate (NBCA) (Histoacryl®, B. Braun, AG, Melsungen, Germany), as the most commonly used adhesive glue, is mixed with lipiodol with a dilution ratio from 1:2 to 1:5 depending on the volume and rate of extravasation. Herein, lipiodol gives the characteristic of X-ray opacity to the mixture, enabling the monitoring of glue embolization; besides, the flexible adjustments of lipiodol/NBCA ratio made the embolization controllable to ensure the complete embolization of the distal leakage site and avoid microcatheter adherence. Before NBCA/lipiodol mixture injection, 5 ml glucose 40% should be used to flush the microcatheter. Once the embolization is accomplished, remove the microcatheter immediately to avoid microcatheter adherence. The following fluoroscopy and CT scan can visualize the glue in the thoracic duct as a linear opacity. Figure 8 presents a case of TDE in a pig model with coils and sequential NBCA/lipiodol mixture embolization.

### Intranodal glue embolization (INE)

In INE, the upstream lymph nodes proximal to the leakage site are punctured using a thin Chiba needle under fluoroscopy or CT guidance. After locating the needle tip in the medulla of the target lymph nodes, the glue is injected to embolize from the efferent lymphatic vessel of the lymph node to the leakage site (Hur, Shin et al. 2016, Smolock et al. 2018, Kim, Hur et al. 2019). Similar to TDE, NBCA is most commonly used as the embolic agent. The dilution ratio of NBCA with lipiodol is usually between 1:4 to 1:6 lower than the ratio in TDE, empirically depending on the extravasating rate and the distance between the accessed lymph node to the leakage site. This treatment is mainly used to treat PLL at the neck, groin, or pelvis because of the short distance between the upstream lymph nodes to the leakage site (Hur, Shin et al. 2016, Smolock et al. 2018, Kim, Hur et al. 2019). The technical success rate of INE was reported as 100%, with a clinical success rate of more than 80% (Hur, Shin et al. 2016, Smolock et al. 2018, Kim, Hur et al. 2019).

**Fig. 9:**
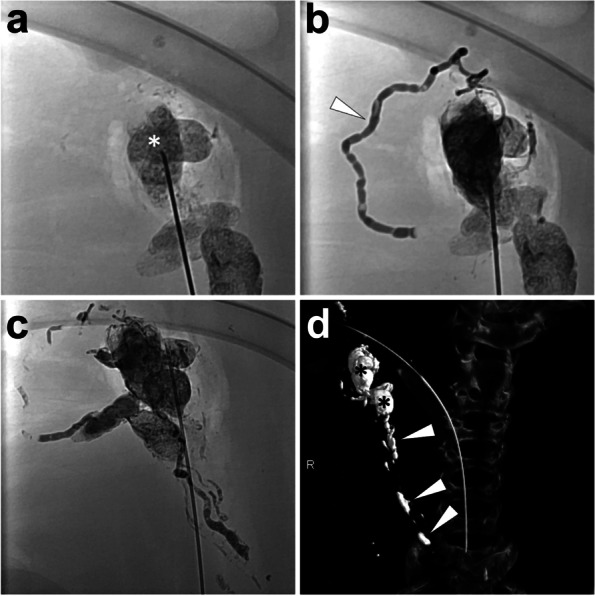
Illustration of cervical INE under fluoroscopy guidance. After prior lipiodol-based lymphangiography, the target lymph node in the right neck (*) was punctured using a 22-gauge Chiba needle under fluoroscopy (**A**). Make the needle tip at the center of the lymph node and remove the stylet; then, inject 1 ml lipiodol through the trocar to confirm the eligible needle position, where the efferent lymphatic vessel (*white arrowhead*) would be gradually visualized but without obvious peripheral extravasation (**B**). After confirmation, inject 4 ml NBCA/lipiodol mixture (1:4) until glue stasis (**C**). Afterwards, the CBCT was implemented to present the spatial details of the embolized lymph nodes (*) and efferent lymphatic vessels (*white arrowhead*s) (**D**). Images were from *Pig No. 10*. Abbreviations: INE, intranodal embolization; NBCA, N-butyl-2-cyanoacrylate; CBCT, cone-beam computed tomography

**Fig. 10:**
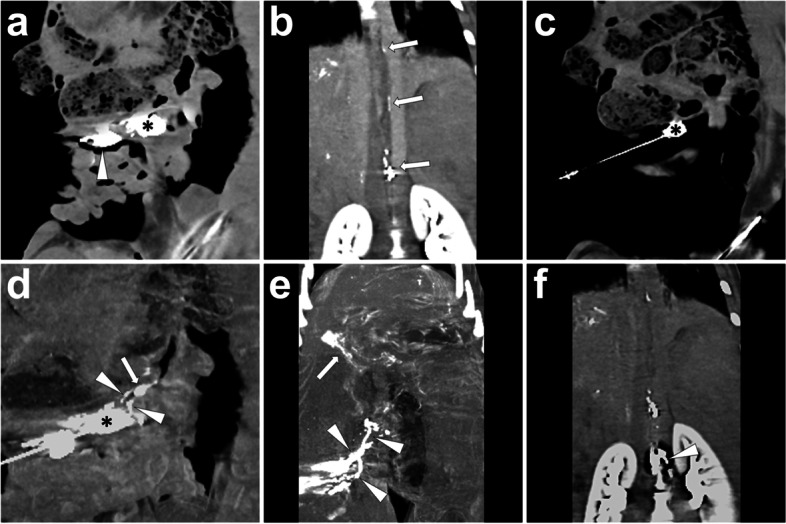
Illustration of mesenteric INE under CT guidance. An hour after prior lipiodol-based laparotomic lymphangiography, the conventional CT was performed, which showed opacified mesenteric lymph nodes [**A**, incl. the cannulated lymph node in the previous lymphangiography (*white arrowhead*) and a surrounding lymph node as the target for following INE procedure (*)] and intermittent opacified thoracic duct (**B**, *white arrows*). In INE procedure, the target lymph node in the mesentery (*) was punctured using a 22-gauge Chiba needle under CT guidance (**C**). Make sure the needle tip is at the center of the lymph node and remove the stylet; then, inject 1 ml lipiodol into the target lymph node (*) to confirm the eligible needle position, where the efferent lymphatic vessels (*white arrowheads*) and distal lymph node (*white arrow*) were visualized but without obvious peripheral extravasation (**D**). After confirmation, inject 4 ml NBCA/lipiodol mixture (1:4); afterwards, the CT was re-implemented to present the embolized efferent mesenteric lymphatic vessels (**E**, *white arrowhead*s) and hepatic lymphatic vessels (**E**, *white arrow*) owing to the glue reflux from embolized cisterna chyli (**F**, *white arrowhead*). Images were from *Pig No. 4*. Abbreviations: INE, intranodal embolization; CT, computed tomography; NBCA, N-butyl-2-cyanoacrylate; CBCT, cone-beam computed tomography

*Materials and Methods.* In our pig model, we can practice INE in lymph nodes in any region. No matter which type of prior lipiodol-based lymphangiography was performed, the lymph nodes in the corresponding area should be opacified under X-ray from previous lipiodol-based lymphangiography. Then, puncture the center of the opacified lymph node under fluoroscopy (Fig.**9**) or CT guidance (Fig.**10**) with a 22-gauge Chiba needle. Then, a small amount (1 to 2 ml) of lipiodol is injected via the trocar to ensure no peripheral extravasation. Noticeably, the water-soluble iodinated contrast is not suggested because it can cause trocar obstruction in the following embolization due to the induction of glue polymerization reaction or precipitation. Once the efferent lymphatic vessel is confirmed, a ratio between 1:4 to 1:6 mixture of NBCA and lipiodol will be sequentially injected through the trocar until glue stasis is achieved. The following CT scan is suggested because it can demonstrate the embolized lymph nodes and efferent lymphatic vessels. Figs. 9 and 10 show cases undergoing INE under fluoroscopy and CT guidance, respectively.

### Percutaneous afferent lymphatic vessel sclerotherapy (ALVS)

Percutaneous ALVS can be used to treat most PLL, such as chylothorax, chylous ascites, and subcutaneous lymphatic leakage, if afferent lymphatic vessels can be identified in prior lipiodol-based lymphangiography (Kortes et al. 2014; Pan et al. 2020a, b, c). In ALVS, the thin needle is punctured as close as possible to the target upstream lymphatic vessels regarding the distal PLL. Then, the sclerosant, such as ethanol, is injected to sclerotize the target afferent lymphatic vessels and surrounding tissue leading to the aseptic inflammation and following occlusion of the leakage (Kortes et al. 2014; Pan et al. 2020a, b, c). The technical success rate of ALVS was reported to be 100%, with a clinical success rate of around 80% (Kortes et al. 2014; Pan et al. 2020a, b, c).

**Fig. 11:**
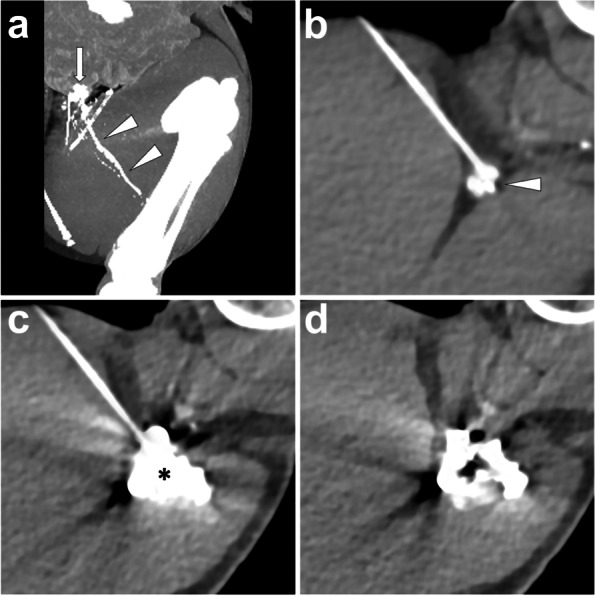
Illustration of percutaneous ALVS. In the PLL pig model, the post-lymphangiography CT scan showed a definite lipiodol extravasation (*white arrow*) at the left groin from the afferent lymphatic vessels (*white arrowheads*) at the left thigh (**A**). After confirming the afferent vessels responsible for the PLL, percutaneously advance a 22-gauge Chiba needle and make the needle tip close to the target lymphatic vessels (*white arrowhead*) at the left thigh (**B**). Inject 2 ml diluted iodinated contrast to show an ideal contrast dispersion completely covering the afferent vessels (*) (**C**). After sequential sclerotherapy by injecting 2 ml of 95% ethanol, remove the needle and carry out the CT scan presenting gas bubbles in the center of the dispersed iodinated contrast (**D**). Images were from *Pig No. 5*. Abbreviations: ALVS, afferent lymphatic vessel sclerotherapy; PLL, postoperative lymphatic leakage; CT, computed tomography

*Materials and Methods.* At first, the lipiodol-based TL with the following post-lymphangiography CT should be performed to identify the definite leakage site and upstream afferent lymphatic vessels. Then, under CT guidance, puncture a 22-gauge Chiba needle to make the tip as close as possible to the afferent lymphatic vessels. Make sure to avoid unnecessary injury to any other sensitive organ or structures. Afterwards, approximately 1 to 4 ml of water-soluble iodinated contrast diluted with normal saline (dilution ratio: 1:10) is injected to observe whether the contrast dispersion completely covers the target lymphatic vessels but without distribution into any other sensitive tissue or structures (e.g., pancreas, adrenal gland, etc.). After confirmation of ideal contrast dispersion, 95% ethanol (B. Braun, Melsungen, Germany) as the sclerosant agent is injected with a dose of less than 5 ml. If multiple afferent lymphatic vessels exist, the procedure should be repeated until all targets are sclerosed. In Fig.11, we illustrate a typical process of ALVS in a PLL pig model.

### Percutaneous afferent lymphatic vessel embolization (ALVE)

Like ALVS, percutaneous ALVE can treat different types of PLL with similar technical and clinical success rates (Baek et al. 2016a, b; Baek et al. 2016a, b; Pan et al. 2020a, b, c). No matter the performance of ALVS or ALVE, the definite afferent lymphatic vessels responsible for PLL should be identified in prior lymphangiography as the sclerosing or embolizing targets. The difference is that in ALVE, the thin needle must be punctured into the efferent lymphatic vessels for the following embolization. Thus, the performance of ALVE is more complicated than ALVS because most peripheral lymphatic vessels are fragile and small. However, from previous studies, a “cutting-off” technique can be suggested: the needle penetrates both the anterior and posterior walls of the afferent lymphatic vessels, and the glue injection is followed along with the needle withdrawal (Baek et al. 2016a, b; Baek et al. 2016a, b; Pan et al. 2020a, b, c). In short, this method can be summarized in two words: disruption and embolization. This technique decreases the operation difficulty with equal efficiency to translymphatic vessel embolization (Baek et al. 2016a, b; Baek et al. 2016a, b; Pan et al. 2020a, b, c).

**Fig. 12:**
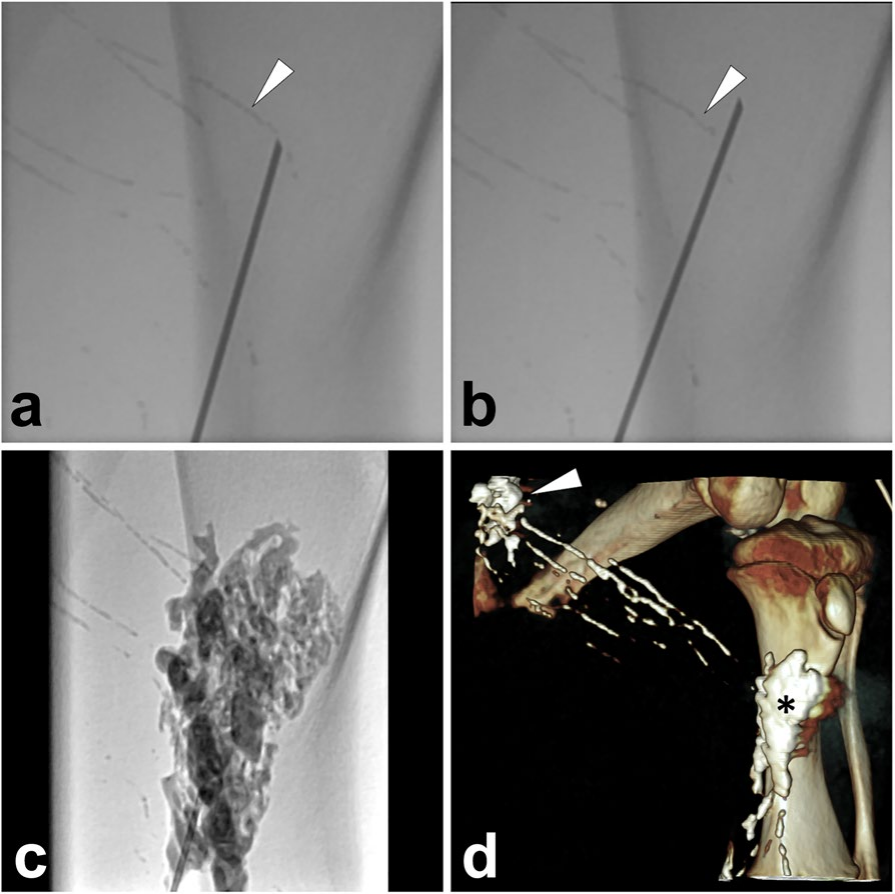
Illustration of percutaneous ALVE using a “cutting-off” technique. In a PLL pig model, puncture the needle targeting the afferent lymphatic vessel (*white arrowhead*) under fluoroscopy (**A**, paralleled flat detector position). Then, cut off the target lymphatic vessel (*white arrowhead*) (**B**, paralleled flat detector position) and inject 1 ml NBCA/lipiodol mixture (1:2) when simultaneously withdrawing the needle. Repeat the same operations to ensure all afferent lymphatic vessels are embolized (**C**). After the ALVE procedure, the volume rendering image of the CT scan demonstrated the relationship between the PLL site (*white arrow*) and the embolized region (*) (**D**). Images were from *Pig No. 1*. Abbreviations: ALVE, afferent lymphatic vessel embolization; PLL, postoperative lymphatic leakage; NBCA, N-butyl-2-cyanoacrylate

*Materials and Methods.* After confirmation of the definite leakage site and upstream afferent lymphatic vessels, percutaneously advance a 22-gauge Chiba needle to cut off the target afferent lymphatic vessel under fluoroscopy with the bull-eye and paralleled flat detector monitoring. Afterwards, the mixture of NBCA/lipiodol (1:2 to 1:4 ratio) is injected with simultaneous needle withdrawal to seal the disrupted lymphatic vessel. Repeat the operation until all afferent lymphatic vessels are embolized. At last, perform the CT scan to identify whether all the target lymphatic vessels are completely embolized. We illustrate an ALVE procedure applying the “cutting-off” technique in Fig. 12.

### Histopathological changes of lymph nodes

**Fig. 13:**
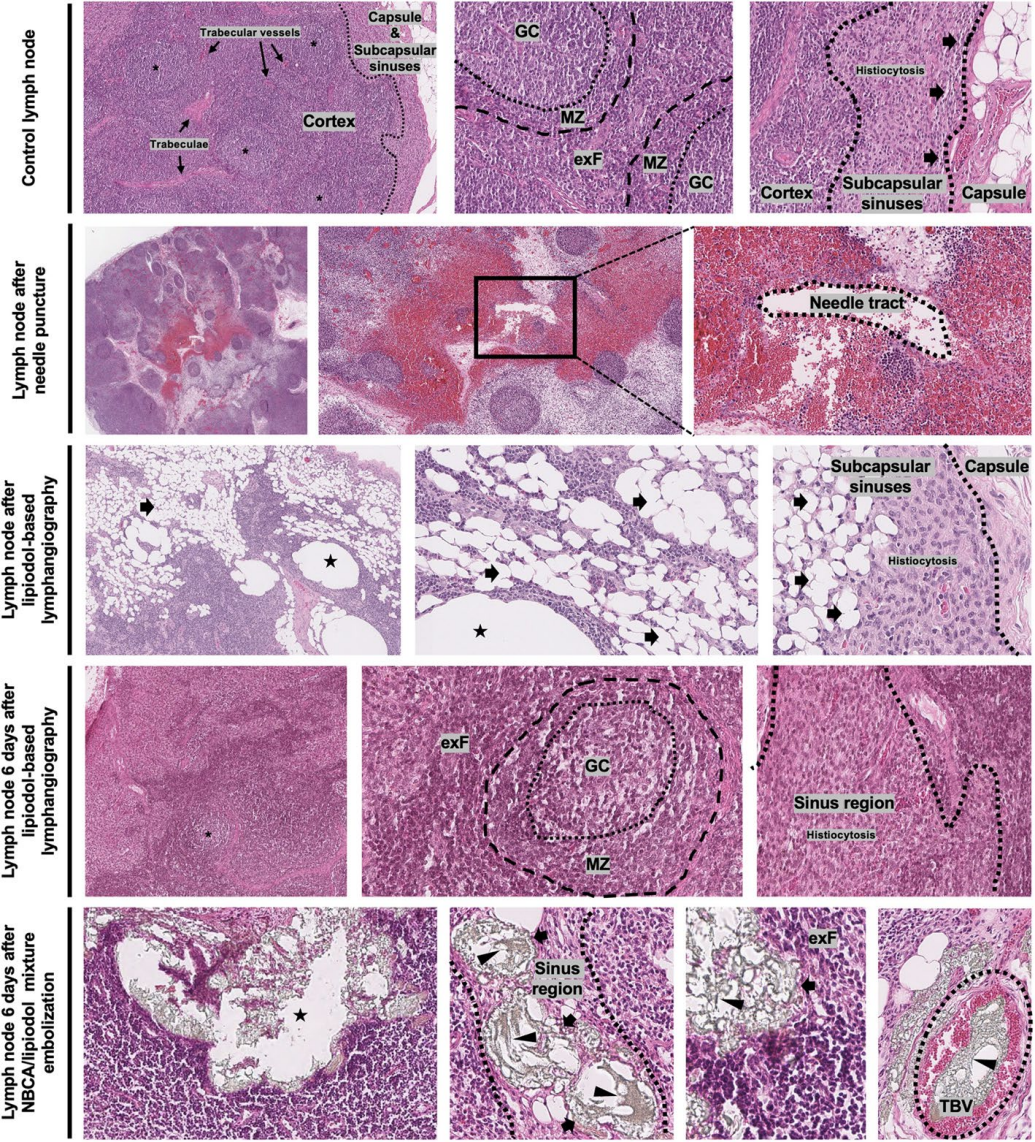
Histopathological demonstrations before and after lipiodol-based INL and INE. The upper row shows an unaffected lymph node structure with outer capsule and subcapsular sinuses, lymphoid follicles (*), and uniform extrafollicular regions (exFs) in the cortex with extended trabeculae and trabecular blood vessels; in each follicle, germinal center (GC) and mantle zone (MZ) are sharply demarcated; besides, there are contact sinuses and moderate histiocytosis beneath the capsule. The second row shows a parenchymal defect of the needle tract with surrounding hemorrhage and focal necrosis. The third row shows extensive empty spaces within the dilated sponge-like sinuses (black arrows) and dilated blood vessels (asterisks) after lipiodol-based INL due to the histological preprocessing. The fourth row shows the restoration of extensive empty spaces without any persistent parenchymal injury 6 days after lipiodol-based INL. The lowest row shows the glue (black arrowhead) injected in INE was seen both within the sinuses (black arrows) and trabecular blood vessels (TBVs), with mildly focal nuclear pyknosis and partial loss of intercellular borders after 6 days. All images were from *Pig No. 7&8* under standard HE stains. Notes: *, lymphoid follicle; asterisk, blood vessel; black arrow, lymphatic sinus; black arrowhead, consolidated glue framework. Abbreviations: GC, germinal center; MZ, mantle zone; exF, extrafollicular zone; TBV: trabecular blood vessels; INL, intranodal lymphangiography; INE, intranodal embolization

Lymph nodes are important sites of antigen presentation, immune activation, and lymphocyte proliferation. They distribute along with the course of the lymphatic vessels to filter the lymph before draining back to the blood circulation. Histologically, completely preserved architectures are seen in unaffected lymph nodes (Fig. 13). These unaffected lymph nodes are encased by a collagenous capsule, which extends into the body of the lymph node as trabeculae. Beneath the capsule, the body of the lymph node is divided into an outer cortex and an inner medulla: the cortex contains many lymphoid follicles and the extrafollicular zones, which are rich in B- and T-cells, respectively; the medulla contains bifurcated sinuses connected with upstreaming cortical and subcapsular sinuses, which finally converge into efferent lymphatic vessels. The follicles are surrounded by regular mantle and marginal zones, sharply demarcated from the extrafollicular zones. Germinal centers are seen within the follicles, which are regularly polarized and, in addition to centrocytes, also contain individual centroblasts. Scattered loosely, there are some tangible-body macrophages. In unaffected lymph nodes, the sinus structures are compact. But in the area of the marginal sinus, moderately increased histiocytes can be observed, corresponding to a sinus histiocytosis. The extrafollicular region is not broadened and essentially contains small lymphocytes, which mostly correspond to T-cells.

After lipiodol-based INL, local hemorrhage can be observed around a sharply defined defect region (indicating the needle tract) in the cortex, probably due to the damage of trabecular blood vessels (Fig. 13). The erythrocytes are distributed diffusely in the adjacent parenchyma. Near the needle tract, a few dissociative parenchymal cells indicate local necrosis. In addition, the application of lipiodol brings characteristic changes within the lymph node. Due to histological preprocessing (incl. fixation, dehydration, and paraffin embedding), fat-like substances such as lipiodol were washed out, leaving optically empty spaces. Such empty spaces are found extensively in the parenchyma with a single endo-epithelial layer, predominantly in the expanded extrafollicular region, indicating deposits of lipiodol in dilated sinuses and blood vessels. Microscopically, they look like a sponge filled with water. However. 6 days later, the lipiodol in the sinuses flowed away or was gradually absorbed with the restoration of the dilated sinuses; parenchymal injury and inflammation were not observed (Fig. 13).

Similar to lipiodol-based INL, 6 days after INE, the sinuses within the lymph node were dilated and filled with lipiodol-glue mixtures within the subcapsular and extrafollicular region, obstructing efferent lymphatic vessels and sinuses; noticeably, ectopic embolization of trabecular blood vessels could also be observed (Fig. 13). Besides, mild parenchymal injury consisting of focal nuclear pyknosis and partial loss of intercellular borders could be found around the embolized lymphatic vessels and sinuses (Fig. 13).

## Discussion

In response to the needs for scientific research and preclinical training, this review summarizes various techniques for lymphangiography in healthy pigs, animal PLL modeling, and different interventional treatments for PLL. As a result, all these presented techniques are proven feasible to perform in living pigs. Besides, we also researched histopathological changes before and after different lymphatic interventions, which helped us to understand the pathophysiological mechanisms behind the lymphatic interventions. In sum, our illustrations aim to teach the following top 3 learning objectives:

1. To get familiar with different access techniques and instruments for lymphatic visualization

This review describes three access routes for lymphangiography: interstitial, translymphatic, and intranodal. The lymphatic capillaries in the interstitial are the initial part of the lymphatic system. Like vascular capillaries, lymphatic capillaries are found in almost all the main organs or tissues, which are surrounded by a thin layer of endothelial cells with loosened connections; so, the macromolecules can pass through the lymphatic wall, thereby realizing lymphatic circulation to regain proteins and other nutrients (Kesler et al. 2013). This histological structure provides a basis for interstitial lymphangiography. Interstitial injection of water-soluble contrast media that can easily permeate into the lymphatic capillaries, such as the dye “Patent Blue V” and gadolinium-based magnetic resonance contrast agents, to achieve the visualization of the lymphatic structures under direct vision or radiological imaging (Mazzei et al. 2017; Pieper et al. 2019; Pieper et al. 2020; Li et al. 2021, Pan, Richter et al. 2022). In this review, we attempted to test the feasibility of lymphangiography by interstitial injection of lipiodol, an oily contrast agent that is difficult to pass the lymphatic wall. As a result, our result showed that the lipiodol in the interstitial could also be absorbed into the lymphatic vessels over time though it is prolonged. Thus, this modality might be more suitable to delineate the lymphatic structures in the upper and lower limbs but not available for the examinations of main lymphatic ducts (e.g., cisterna chyli, thoracic duct, etc.). In the translymphatic route, the large lymphatic vessels can be precisely cannulated after incising the skin and separating the stained lymphatic vessels at the distal limbs (Pieper et al. 2019). However, this method is technically challenging and time-consuming. Therefore, the intranodal route becomes a more acceptable approach. In previous clinical reports, ultrasonography is the mainly used guidance for intranodal cannulization (Nadolski and Itkin 2012). However, this review provides two other possible methods of lymph node puncture: 1. Surgical exposure for direct-vision puncture. This method has been reported earlier in experiments on rabbits (Matsumoto et al. 2019). This review used this modality for mesenteric INL, and it worked well. This could provide a new research method to understand the mesentery's lymphatic drainage system further. 2. Bridging percutaneous lymph node puncture under fluoroscopy. After previous lipiodol-based lymphangiography, the lymph nodes can be opacified. Then, under fluoroscopy guidance, the target lymph node can be precisely punctured for further lymphangiography. This approach can be used for short-term re-examination after the lipiodol-based lymphography or when the main lymphatic ducts were not well opacified in the previous lymphangiography.

Different imaging methods can be used depending on the type of contrast agent injected. For lipiodol-based lymphangiography, fluoroscopy, DSA, conventional CT, and CBCT can be chosen to visualize the lymphatic system in the corresponding examination area (Pan et al. 2020a, b, c; Sommer et al. 2021). Fluoroscopy and DSA, as two-dimensional imaging techniques, cannot provide the three-dimensional spatial information of the lymphatic system and the surrounding neighbor structures, but they can realize dynamic monitoring of lymph flow and make it easier to observe the leakage site of PLL. Although traditional CT or CBCT is difficult to achieve dynamic monitoring, it can provide three-dimensional anatomy information and has better sensitivity for PLL detection, which is helpful for subsequent lymphatic intervention planning (Pan et al. 2020a, b, c). For gadolinium-based MRL, three-dimensional gradient-echo such as 3D-FLASH is the most commonly used imaging sequence (Notohamiprodjo et al. 2012; Mitsumori et al. 2015; Mazzei et al. 2017; Pieper et al. 2020, Ramirez-Suarez, Tierradentro-Garcia et al. 2021, Smith, Liu et al. 2021). However, the timing of MRI scans after the injection of interstitial contrast agent remains unclear because the flow rate of contrast agent into the lymphatic circulation varies greatly, especially in pathological conditions; in addition, there is a remarkable difference in the development time of lymphatic vessels at different areas. To standardize the MRL scanning time, we provided a feasible strategy: first, immediately after the interstitial contrast agent injection, a 3D-TWIST rapid scanning sequence [a time-resolved 3D-MRI angiography technique with very high temporal (sub-second) and spatial resolution (sub-millimeter) based on unique *k*-space sampling to accelerate the scanning] was performed to monitor the development of lymphatic vessels in the region of interest; then, once the 3D-TWIST presented clear visualization of the lymphatic vessels at the examined area, the 3D-FLASH scan was implemented to show fine anatomical details with high-quality images. This method can improve the technical success rate of the MRL examination.

2. To learn about different lymphatic interventions for PLL

This review presents four common interventional treatments for PLL. Whichever approach is taken, embolization or sclerosis of the responsible lymphatic vessels for the PLL is necessary (Pieper et al. 2019; Pan et al. 2020a, b, c; Sommer et al. 2021). The treatment principles include: 1. Complete embolization or sclerosis of the responsible lymphatic vessels; 2. The embolization or sclerosis should be as close to the leakage site as possible (Pieper et al. 2019; Pan et al. 2020a, b, c; Sommer et al. 2021). In TDE, one of the most commonly used translymphatic embolization treatments, the operator usually punctures the chyle cistern to coaxially advance a microcatheter to the thoracic duct for embolization (Cope and Kaiser 2002, Boffa et al. 2008, Mittleider et al. 2008, Itkin, Kucharczuk et al. 2010, Yannes et al. 2017, Majdalany et al. 2018, Reisenauer et al. 2018, Jun, Hur et al. 2021). Because the diameter of the cisterna chyli is relatively large (4–6 mm) and the path from the cisterna chyli to the thoracic duct is a single-channel structure if without anatomical variation, the percutaneous trans-cisterna chyli cannulization and thoracic duct microcatheterization become possible. However, this method is unsuitable for embolizing peripheral lymphatic vessels, which are too thin. Two interventional techniques can be used for the embolization of peripheral lymphatic vessels: INE and ALVE (Baek et al. 2016a, b, Baek et al. 2016a, b, Hur, Shin et al. 2016, Smolock et al. 2018, Kim, Hur et al. 2019, Pan et al. 2020a, b, c). INE is an embolization technique developed based on INL, which can effectively embolize downstream lymphatic vessels. However, it should be noted that INE has high requirements for the operator's experience in using liquid glue because the concentration and injection velocity of the glue may affect the embolization effect on the target lymphatic vessels. Although this review provides the recommended concentration of NBCA glue, the operator still requires flexible adjustments according to the specific situation to achieve the eligible embolization of the responsible lymphatic vessel closest to the leakage site. In addition, ALVE provides another feasible method of lymphatic embolization. Compared with INE, this method requires relatively high puncture. In INE, we only need to place the needle tip in the central area of the opacified lymph node, while in ALVE we need to accurately puncture the needle into the target lymphatic vessel (Baek et al. 2016a, b; Baek et al. 2016a, b; Pan et al. 2020a, b, c). Even applying the "cutting-off" technique, penetration of the target lymphatic vessels is necessary. Therefore, this method may be more suitable for two clinical scenarios: 1. Lack of afferent lymph nodes close to the PLL site (e.g., PLL in upper and lower limbs, etc.); 2. Safe puncture path (e.g., low risks of accidental injury to the aorta and ectopic vascular embolism, etc.). As the only sclerotherapy we illustrated, ALVS is relatively less technically challenging. It is only necessary to place the needle tip as close as possible to the responsible lymphatic vessels. However, sclerotherapy can cause aseptic inflammation of the tissue, which is also destructive, so before sclerotherapy, it is essential to carefully evaluate whether there are sensitive tissues or organs, such as nerves and blood vessels around the target lymphatic vessels. Therefore, injecting a small amount of diluted water-soluble iodinated contrast agent is recommended to estimate the diffusion area under CT guidance, which can help predict the injection dosage of sclerosant and treatment risk.

3. To recapitulate key anatomical and histopathological features relevant before and after lymphangiography and lymphatic interventions

So far, there have been few reports of histopathological changes in lymph nodes after lipiodol-based lymphangiography and lymphatic interventions. Other studies have suggested that lipiodol might lead to local inflammation benefiting tissue proliferation (Lv et al. 2017; Pieper et al. 2019; Pan et al. 2020a, b, c; Sommer et al. 2020, Pan, Richter et al. 2022). However, this study found that lipiodol did not cause any injury or inflammation to the surrounding lymph node parenchyma; after entering the lymphatic sinuses, lipiodol was gradually absorbed or flowed away over time, restoring a contact and unaffected lymph node structure. Therefore, this study demonstrates the non-toxicity of lipiodol application in the lymphatic system from a histopathological point of view. It can be inferred that for the therapeutic effect of lipiodol-based lymphangiography for post-surgical lymphatic fistulas, lymphatic embolization by lipiodol is most likely to be the main reason.

In addition, after IDE treatment, the extensive lymphatic sinuses are filled with the lipiodol-glue mixture. Microscopically, the glue forms a frame structure in which the lipiodol fills, resulting in the blockage of the lymph flow. However, it is essential to note that some of the embolic agents enter the blood vessels in the parenchyma, suggesting that there may be potential lymphovascular connections within the lymph nodes. As a shred of additional evidence, the present study also found abnormally dilated blood vessels in the lymph node parenchyma presenting as larger empty spaces in the histopathological examination of lymph nodes after INL, suggesting the fact that lipiodol entered the blood circulation through the lymph sinuses. These findings partly explain why in inguinal iodine-based INL, there is a reported risk of ectopic embolism due to potential lymph-venous shunts (Hsu and Itkin 2016; Jardinet et al. 2021). Although the lipiodol-glue embolization may lead to injury to the parenchyma around lymphatic sinuses, the damage is very mild and focal. Therefore, IDE can still be considered as a safe lymphatic interventional modality.

This review also has limitations. First, we summarized three access approaches for lymphangiography with two types of contrast applications but without a controlled or interaction study design because some pigs were occasionally unsuitable for specific lymphatic interventions. For instance, the small cisterna chyli suggested an inappropriate candidate for TDE; thin lymphatic vessels at extremities indicated the infeasibility of translymphatic lymphangiography and interventions. After all, the primary purpose of this review was to illustrate clinically used lymphography techniques in pigs. Secondly, although we have summarized a variety of interventional treatments, all these methods are used for PLL, and only commonly used embolic and sclerosing agents have been explored. Third, our PLL establishment is currently limited to the groin area. For other clinical types of PLL, such as chylothorax and chyle ascites, we have not found an optimal modeling method. So, various lymphatic interventions were mainly carried out in imaginary scenarios of different PLLs. Finally, there are rarely used lymphatic interventions that we have not mentioned in this review, such as retrograde thoracic duct embolization, transhepatic lymphatic embolization, etc., which merit further research in animals (Mittleider et al. 2008; Hasegawa et al. 2021). In addition, translymphatic and intranodal MRLs are also worth exploring.

## Conclusion

This pictorial review presents various types of lymphangiography and lymphatic interventions that utilize the German landrace pig model. It has the potential to facilitate the standardization of preclinical training, allowing inexperienced radiologists and interventional radiologists, such as fellows and residents, to acquire practical skills in these techniques through training on pigs. Besides, our summarized presentation also provides feasible experimental methods for different lymphatic research purposes.

## Data Availability

All data were illustrated in this article and no additional data were available.

## Abbreviations

3D-FLASH: Three-dimensional fast low angle shot
3D-TWIST: Three-dimensional time-resolved angiography with interleaved stochastic trajectories
ALVE: Afferent lymphatic vessel embolization
ALVS: Afferent lymphatic vessel sclerotherapy
CBCT: Cone-beam computed tomography
CT: Computed tomography
DSA: Digital subtraction angiography
exF: Extrafollicular zone
GC: Germinal center
HE: Hematoxylin and eosin
INE: Intranodal embolization
INL: Intranodal lymphangiography
MIP: Maximum intensity projection
MRI: Magnetic resonance imaging
MRL: Magnetic resonance lymphangiography
MZ: Mantle zone
NBCA: N-butyl-2-cyanoacrylate
PLL: Postoperative lymphatic leakage
TBV: Trabecular blood vessels
TDE: Thoracic duct embolization
TE: Echo time
TL: Translymphatic lymphangiography
TR: Repetition time
US: Ultrasonography
